# Access to head and neck cancer specialists: a geospatial analysis of U.S. travel time

**DOI:** 10.3389/fonc.2025.1521370

**Published:** 2025-07-31

**Authors:** Bradley L. Goodnight, Glenn J. Hanna, Dandan Zheng, Magdiel Habila, Marie Cassese, Alexander Fortman, Harold Walbert, Fred Sieling, Christopher M. Black

**Affiliations:** ^1^ Health Data Science, Guidehouse Inc., Tysons, VA, United States; ^2^ Center for Head & Neck Oncology, Dana-Farber Cancer Institute, Boston, MA, United States; ^3^ Outcomes Research, Merck & Co., Inc., Rahway, NJ, United States

**Keywords:** geographic disparities, head and neck cancer, health equity, social determinants of health, access to care, disparity index, travel time analysis

## Abstract

**Introduction:**

Head and neck (H&N) cancers, a diverse group of epithelial malignancies, significantly impact patients' quality of life and require complex, multidisciplinary care. Despite the need for specialized care, access to H&N cancer specialists is uneven across the United States, leading to disparities in patient outcomes and health equity. To assess geographic disparities in access to H&N cancer specialists in the U.S. and to identify factors contributing to these disparities, with the goal of informing targeted interventions and policies that promote equitable healthcare access.

**Methods:**

This geospatial analysis utilized data from various public databases, including the National Provider Identifier Registry, American Society of Clinical Oncology, and U.S. News and World Report, to examine the distribution of H&N cancer specialists relative to incident cancer cases. The study analyzed county-level data across the United States, incorporating demographic factors such as race/ethnicity, age, education, and socioeconomic status. Travel time to the nearest H&N specialist was estimated using the Travel Time API. The analysis included 1,112 H&N specialists (453 surgical oncologists, 346 medical oncologists, and 308 radiation oncologists) identified through self-reported data and relevant fellowships. The primary outcome was the estimated travel time to the nearest H&N specialist, with secondary measures including a disparity index that combined cancer incidence, social vulnerability, and travel time to highlight regions with the greatest access disparities.

**Results:**

Significant regional disparities in access to H&N specialists were identified, with non-metropolitan areas and regions outside the Northeast showing notably longer travel times. Socioeconomic and demographics factors, including lower household income, lower insurance coverage, and higher median age, were associated with increased travel times. Disparity Index scores highlighted counties in the South and Western regions as having the highest access disparities.

**Conclusion:**

Geographic and socioeconomic disparities in access to H&N cancer specialists contribute to health inequities in the U.S. The disparity index developed in this study provides a valuable tool for identifying high-need areas and guiding policy interventions. Addressing these disparities through targeted resource allocation, mobile clinics, and provider incentives is essential for improving access to specialized care and promoting health equity.

## 1 Introduction

Head and neck (H&N) cancers represent a diverse and challenging group of epithelial malignancies that target complex anatomical structures comprised of the larynx, pharynx, nasal cavity, paranasal sinuses, and oral cavity (1–4). Worldwide, H&N cancer ranks as the seventh most common type of cancer (5). While tobacco, alcohol use and occupational exposures are traditional risk factors, human papillomavirus (HPV) infection-related carcinomas are on the rise (1, 3, 5, 6). Both the cancer and its treatment can affect essential functions, such as speech, swallowing, and respiration, which has a profound impact on the patient’s quality of life (2). As such, treatment goals aim to improve survival and to preserve function.

Due to the complexity of the disease and potential impact on patients, optimal care planning and management of H&N cancer should involve collaboration among a range of healthcare professionals with relevant expertise via a multidisciplinary team, or MDT (2, 7). Governing organizations such as the European Society for Medical Oncology (ESMO), the National Comprehensive Cancer Network® (NCCN®), the Society of Medical Oncology (SEOM), and the American College of Surgeons (ACS) recommend that treatment plans be established by an MDT (8–11). Treatment plans for H&N cancers may require surgery, radiation, chemotherapy, targeted therapy, and supportive care, depending upon the location and stage of disease (2, 4, 7). Patients with H&N cancers often present with locoregionally advanced disease, and surgery, radiotherapy, and/or chemotherapy is the initial treatment in many of these patients (12, 13). Access to this specialized care is pivotal in determining the trajectory of a patient's journey from diagnosis to survival (14, 15). The complex treatment modalities required for H&N cancers, including intricate surgical procedures and targeted radiation therapy delivery and techniques, necessitate specialized knowledge and experience (1–4, 11, 16). These patients may also develop complications associated with their disease and treatments, which can lead to physical, social, and psychological problems. Careful monitoring and participation in supportive care programs are important to maintain these patients’ quality of life (17).

However, in the United States access to specialized cancer care is not equally available for all patients, particularly in rural areas and in the West and Midwest regions (18, 19). These disparities are rooted in a complex web of factors, including socioeconomic status, racial and ethnic background, and healthcare infrastructure (20–24). H&N cancers are impacted by disparities in access to specialized care, which results in disparities in individual patient outcomes and the perpetuation of health inequities (25–28). Specifically, limited access to H&N cancer specialists could potentially result in delayed diagnoses, suboptimal treatment, and poorer outcomes, as well as reduced survival rates and quality of life (25, 29). A deeper understanding of disparities based on location and social determinants of health can drive the development of targeted interventions, improvement healthcare policies, and innovative healthcare delivery models that mitigate the impact of inequities (21, 27, 29).

To help improve the survival prospects and quality of life for individuals confronting H&N cancers while contributing to the broader mission of reducing healthcare disparities in the U.S. a comprehensive exploration of disparities in geographic access to H&N cancer specialists across the United States was conducted. The aims of the current study are to advance dialogue on healthcare disparities and advocate for equitable, patient-centered cancer care that transcends geographical and social boundaries. Geospatial analysis was used to assess the impact of factors, such as. This study tested the hypotheses that U.S. region, metropolitan status, socio-economic factors, and cancer epidemiology significantly contribute to disparities in access to H&N cancer specialists. By shedding light on these disparities, this study provides valuable insights for clinicians, policymakers, and researchers to develop strategies that promote equitable access to H&N cancer specialized care.

## 2 Materials and methods

### 2.1 Study design

This geospatial analysis examines the geographic distribution of H&N cancer specialists and incident cancer cases at the state and county levels for larynx and oral cavity/pharynx sites. It assesses demographic factors such as race/ethnicity, age, gender, education, insurance coverage, and employment status using secondary data.

### 2.2 Data sources

**Figure 1:**
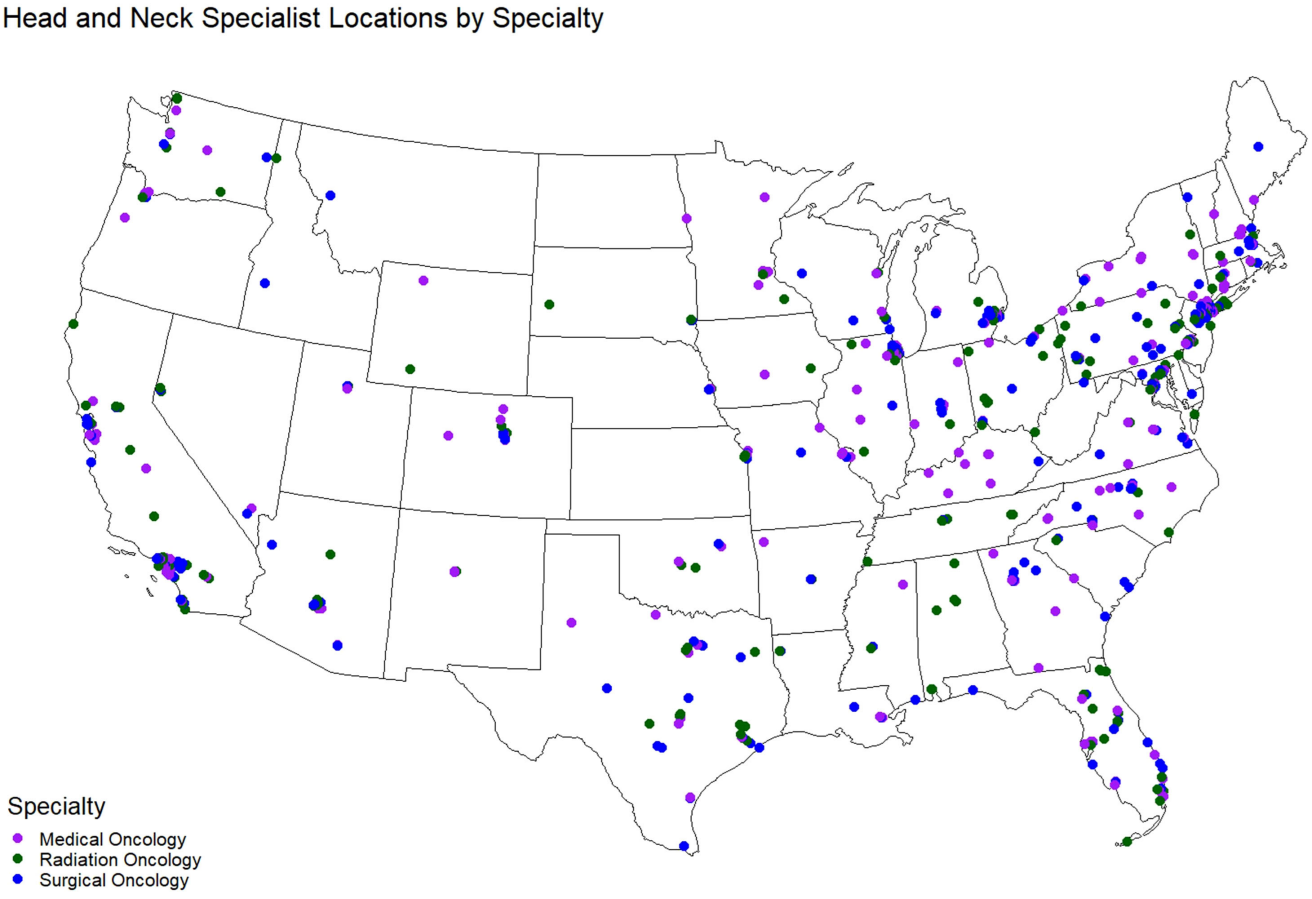
Map of H&N specialists in the U.S.

Data on H&N providers were sourced from the National Provider Identifier Registry (NPI/NPPES), American Society of Clinical Oncology (ASCO), and US News and World Report Doctor Finder. H&N specialists included those with self-reported primary specialties in medical, radiation, or surgical oncology and reported H&N cancer as a subspecialty. Specialists were matched to unique entries in the NPPES NPI Registry and H&N cancer care fellowships. While several types of providers may provide H&N cancer care, such as clinical social workers, physicians, and nurses, we included only H&N cancer oncologists that had a specialization within the taxonomy codes listed in **Table 1**, and further limited our list of H&N specialists to those providers that self-identified as H&N specialists either by: 1) being listed as a H&N specialist in the ASCO database, or 2) by listing H&N oncology (combination of “head and neck” with “cancer” or “oncology/oncologist”) as a specialization among their list of specialties in the US News Doctor Finder. Otolaryngologists (ENTs) in the US News dataset were further reduced to include only H&N specialists that completed a fellowship in a field related to oncology or H&N-related surgery (**Table 2**). Final distribution of specialists by type is shown in **Figure 1**.

**Table 1:** List of taxonomy codes used to identify potential head and neck specialists.

Code	Specialty	Row Count
Starts with “207Y”	Otolaryngology	19486
207RX0202X	Internal Medicine - Medical Oncology	7132
207RH0003X	Internal Medicine - Hematology & Oncology	18253
1223S0112X	Dentist - Oral and Maxillofacial Surgery	13709
1223X0008X	Dentist - Oral and Maxillofacial Radiology	288
204E00000X	Oral & Maxillofacial Surgery	2403
2086X0206X	Surgery - Surgical Oncology	2824
2085R0001X	Radiation Oncology	8899
2085N0700X	Radiology – Neuroradiology	3936

**Table 2:** List of ENT fellowships from US news data with inclusion / exclusion status.

Included	Fellowship	US News Count	Included	Fellowship	US News Count
Yes	Head & Neck Surgical Oncology/Microvascular Reconstruction	330	No	Forensic Pathology	1
Yes	Otolaryngology - Head and Neck Surgery	50	Yes	Head & Neck Oncology, Facial Plastic & Reconstructive Surgery	1
No	Facial Plastic & Reconstructive Surgery	47	Yes	Head & Neck Reconstruction, Microvascular Surgery	1
Yes	Rhinology and Skull Base Surgery	23	Yes	Head & Neck Surgery, Tranoral Robotic Surgery	1
Yes	Complex General Surgical Oncology	7	Yes	Head & Neck Surgery, Microvascular reconstruction	1
No	Pediatric Otolaryngology	7	Yes	Head & Neck Surgical Oncology and Microvascular Reconstruction	1
Yes	Laryngeal Surgery	6	Yes	Head & Neck, Microvascular and Reconstructive Surgery	1
Yes	Laryngology	6	Yes	Head and Neck	1
Yes	Neurotology	6	Yes	Head and Neck and Cranial Base Surgery	1
Yes	Head and Neck Oncologic Surgery	3	Yes	Head and Neck Oncologic Surgery and Reconstructive Microsurgery	1
Yes	Head and Neck Surgery	3	Yes	Head and Neck Oncology	1
Yes	Cancer Biology	2	Yes	Head and Neck Surgery and Microvascular Reconstruction Fellowship	1
Yes	Cranial Base Surgery	2	Yes	Head and Neck Surgical Oncology	1
No	Facial Plastic and Reconstructive Surgery	2	Yes	Head and Neck Surgical Oncology and Microvascular Reconstructive Surgery	1
Yes	Head & Neck Surgical Oncology	2	Yes	Head and Neck/Skullbase	1
Yes	Hematology and Medical Oncology	2	No	Health Policy and Outcomes	1
Yes	Reconstructive Microsurgery	2	No	Hospice and Palliative Medicine	1
No	Sleep Medicine	2	Yes	Laryngology, Voice Disorders, & Bronchoesophagology	1
Yes	Thyroid and Parathyroid Surgery	2	Yes	Laryngology/Neurolaryngology	1
No	Unknown	1	No	Medical Ethics	1
No	AAO-HNSF Humanitarian Fellow	1	Yes	Micrographic Surgery and Dermatologic Oncology	1
Yes	Advanced Head and Neck Surgical Oncology and Microvascular Reconstruction	1	Yes	Microvascular Plastic Surgery	1
Yes	Cancer	1	Yes	Microvascular Reconstructive Surgery	1
Yes	Cancer Bioengineering	1	No	Molecular Epidemiology	1
Yes	Cancer cell biology	1	Yes	Molecular Oncology	1
Yes	Cancer Genetics	1	No	Neurobiology	1
Yes	Clinical Cancer Research	1	No	Neurologic Disease and Blindness	1
No	Clinical Research	1	No	Oral & Maxillofacial Surgery	1
No	Craniomaxillofacial Surgery	1	No	Otology Research	1
Yes	Endocrine Head & Neck Surgery	1	Yes	Skull Base Oncologic Surgery/Head and Neck Surgery	1
No	Experimental Pathology	1	Yes	Skull Base/Facial Plastics	1
No	Facial Plastic Surgery	1	No	Surgical Endocrinology	1
No	Facial Plastics	1	No	Translational Cancer Research	1
Yes	Facial Plastics/MOHS Skin Cancer	1	Yes	Tumor Immunology	1

US population data were obtained from the US Census and CDC Social Vulnerability Index, which combines 16 census variables to measure community-level risk and vulnerability (30). Cancer incidence was obtained from CDC’s United States Cancer Statistics (USCS). H&N cancer incidence was measured for the following cancer sites: 1) larynx, 2) oral cavity and 3) pharynx. All data sources underwent thorough cleaning and integration to ensure consistency and reliability.

### 2.3 Travel time estimation

**Figure 2:**
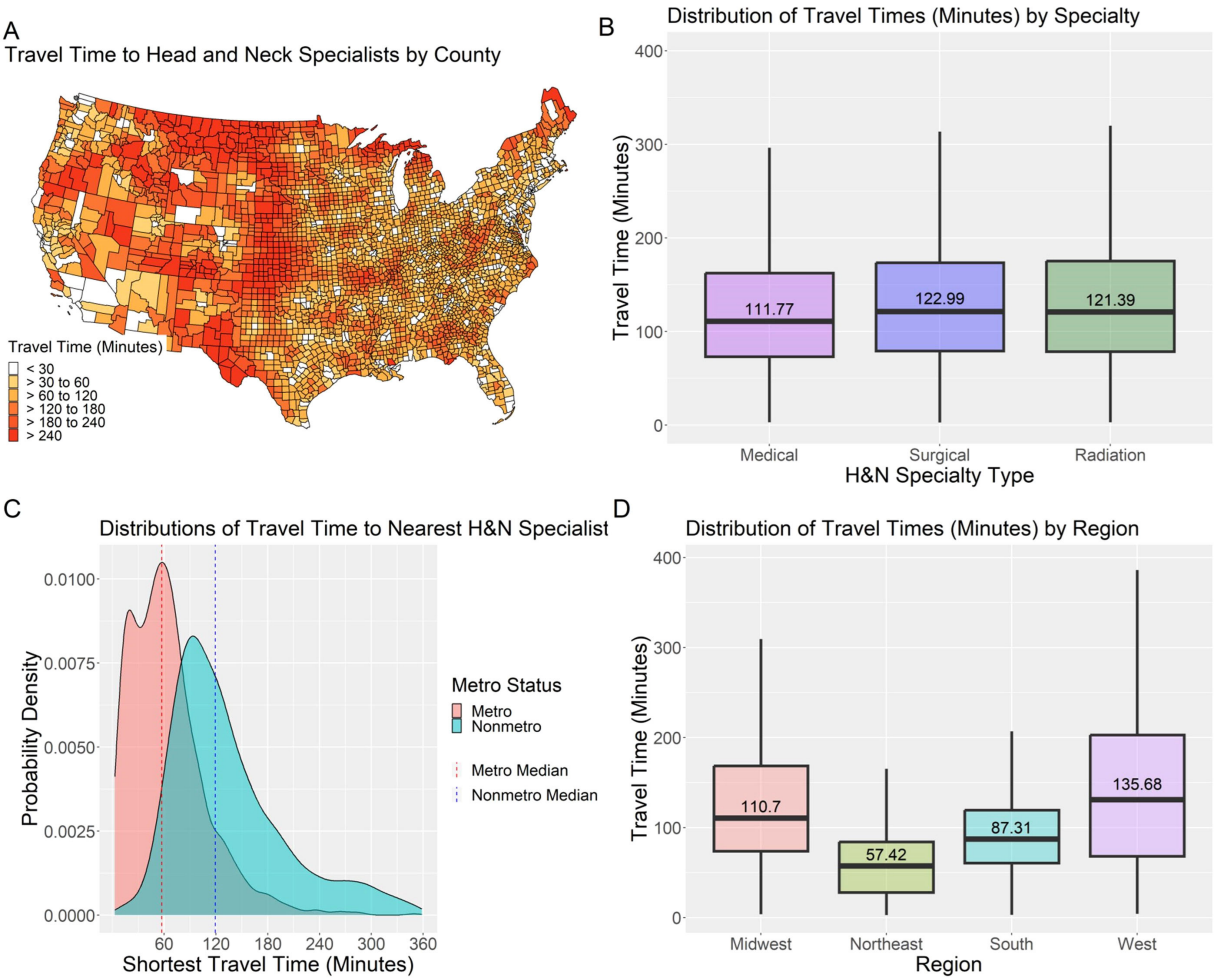
Distributions of travel time to the nearest H&N specialist by US county, provider specialty, county metro status, and US region: **(A)** isochrone map of travel time to nearest head and neck cancer specialist from population-weighted county centroid; **(B)** box plot of access metric by head and neck specialist type; **(C)** probability density function of county-level drive-time to nearest head and neck cancer specialist by metro status; and **(D)** box plots of access metric by U.S. Census region.

Travel time in minutes was estimated from US county population-weighted centroids to nearest H&N specialist using the Travel Time API (31). This utilized road network data and considered various factors affecting travel time. All estimates assumed a starting time of 9:00AM local time for the county centroid. TravelTime offers options for walking, biking, and public transportation, although analyses indicated that travel by car was consistently the fastest mode of transportation. The fastest available mode of transportation by location was used in all analyses. Travel time estimation could only return values under twelve hours (720 minutes), and counties more than twelve hours from the nearest H&N specialist were excluded from analyses. **Figure 2** shows travel time to the nearest H&N specialists geographically, by metro status, region, and specialist type.

### 2.4 Statistical analysis

**Figure 3:**
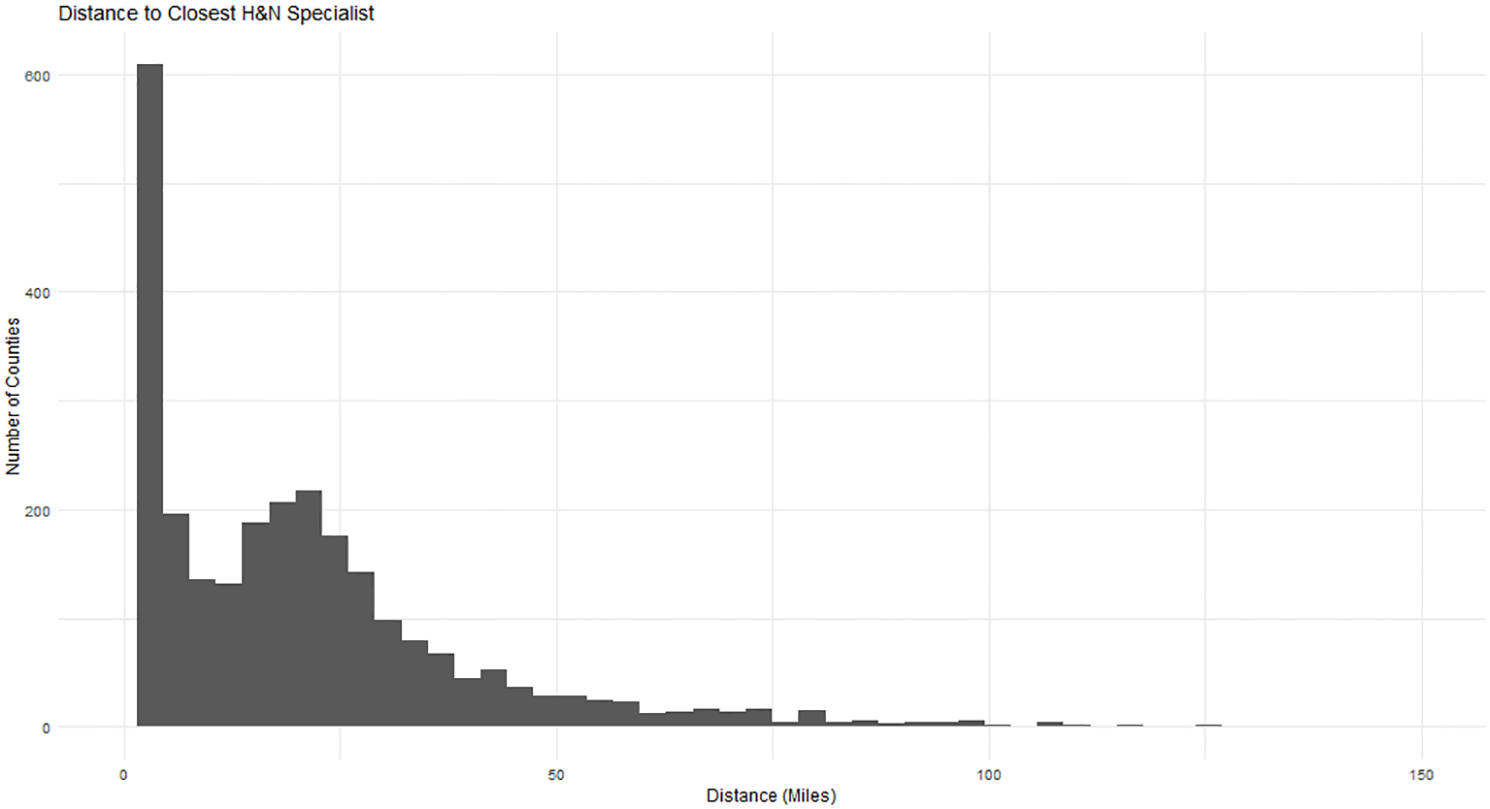
Histogram of travel time to the nearest H&N specialist from county population-weighted centroids.

The analysis consisted of descriptive and visual inspections of travel time distributions and social determinants of health variables from the SVI. Social determinants of health (SDOH) variables included in the analysis were the median age and income of all county residents, and the percent of the county population that were male, white, Hispanic, employed, had health insurance, or had a high-school diploma. Other SDOH variables from the SVI were excluded to avoid multicollinearity. Median travel time by U.S. region and metropolitan (metro) and non-metropolitan (non-metro) status was analyzed. Pairwise differences in accessibility in regions were compared using Pairwise Wilcoxon Tests (**Table 3**). Multivariate quantile regression analysis was performed to assess the relative effects of SDOH and cancer incidence on median estimated travel time across specialist types. Quantile regression was selected over standard linear regression due to the observed skewness in travel time (**Figure 3**). The model included a suppression flag control variable to account for the effect of suppressed values for cancer incidence (mortality counts below 16 in the USCS data) in the model. Data cleaning, integration, and statistical analyses were conducted using R version 4.3.1.

**Table 3:** Pairwise Wilcoxon tests for differences in access by US region.

Region 1	Region 2	Pairwise Wilcoxon tests with Bonferroni correction (*p*-value)
Northeast Region	Midwest Region	<.001
South Region	Midwest Region	.016
West Region	Midwest Region	.702
South Region	Northeast Region	<.001
West Region	Northeast Region	<.001
West Region	South Region	1

### 2.5 Disparity index

A disparity index for H&N specialist access at the county-level was developed, combining H&N cancer incidence, SVI, and estimated travel time to the nearest specialist. The disparity index is a linear combination of county-level access to H&N specialists, cancer incidence, and social vulnerability, which highlights areas where need for care (measured by cancer incidence and SVI) is high and access to care (measured by minimum travel time to the nearest H&N specialist) is low. The index is visualized as a choropleth showing the distribution of access disparity across the US allowing for identification of counties with highest and lowest disparity (**Table 4**) in terms of access to care (travel time), need for care (cancer incidence), and barriers to care (social vulnerability). Each of the three factors were weighted equally using percentile rank in the calculation. A reliability assessment was performed using Cronbach’s alpha to assess the level of internal consistency between all items in the SVI, H&N cancer incidence rates, and estimated travel time.

**Table 4:** Highest and lowest counties by disparity index and metro status.

County	State	Metro Status	Disparity Index Score	Minimum Travel time (Minutes)	Cancer Rate (Per 100k)	SVI Total (Percentile Rank)
Ten US Counties with Highest Disparity Index Scores						
Franklin	Florida	Nonmetro	0.93	252.40	19.33	91%
Calhoun	Georgia	Nonmetro	0.92	165.07	22.10	95%
Alexander	Illinois	Metro	0.92	175.37	23.70	92%
Dimmit	Texas	Nonmetro	0.91	143.35	21.10	100%
Quitman	Georgia	Nonmetro	0.91	206.78	22.20	84%
Cottle	Texas	Nonmetro	0.90	175.57	23.20	87%
Gulf	Florida	Metro	0.85	238.17	19.33	67%
Potter	Texas	Metro	0.82	118.68	16.10	98%
Bay	Florida	Metro	0.80	145.47	17.40	73%
Josephine	Oregon	Metro	0.80	192.83	15.70	71%
Ten US Counties with Lowest Disparity Index Scores						
Scott	Illinois	Nonmetro	0.14	71.38	8.60	2%
Williams	Ohio	Nonmetro	0.13	20.05	10.40	13%
Preble	Ohio	Nonmetro	0.13	49.93	10.50	4%
Hanson	South Dakota	Nonmetro	0.12	69.98	8.15	0%
Lincoln	South Dakota	Metro	0.09	29.10	9.50	4%
Broomfield	Colorado	Metro	0.09	15.18	10.00	7%
Howard	Maryland	Metro	0.09	12.12	8.90	14%
Calumet	Wisconsin	Metro	0.09	58.03	8.10	1%
Johnson	Kansas	Metro	0.08	6.55	9.90	9%
Park	Wyoming	Nonmetro	0.06	26.95	6.20	7%

## 3 Results

### 3.1 Classification of specialist types

The combination of ASCO and US News data sources captured 1,112 H&N specialists who all had active NPI records.

The final sample consisted of 453 surgical oncology H&N specialists, 346 medical oncology H&N specialists, and 308 radiation oncology H&N specialists (**Figure 1**).

### 3.2 Factors associated with travel time to the nearest H&N specialist

**Figure 4:**
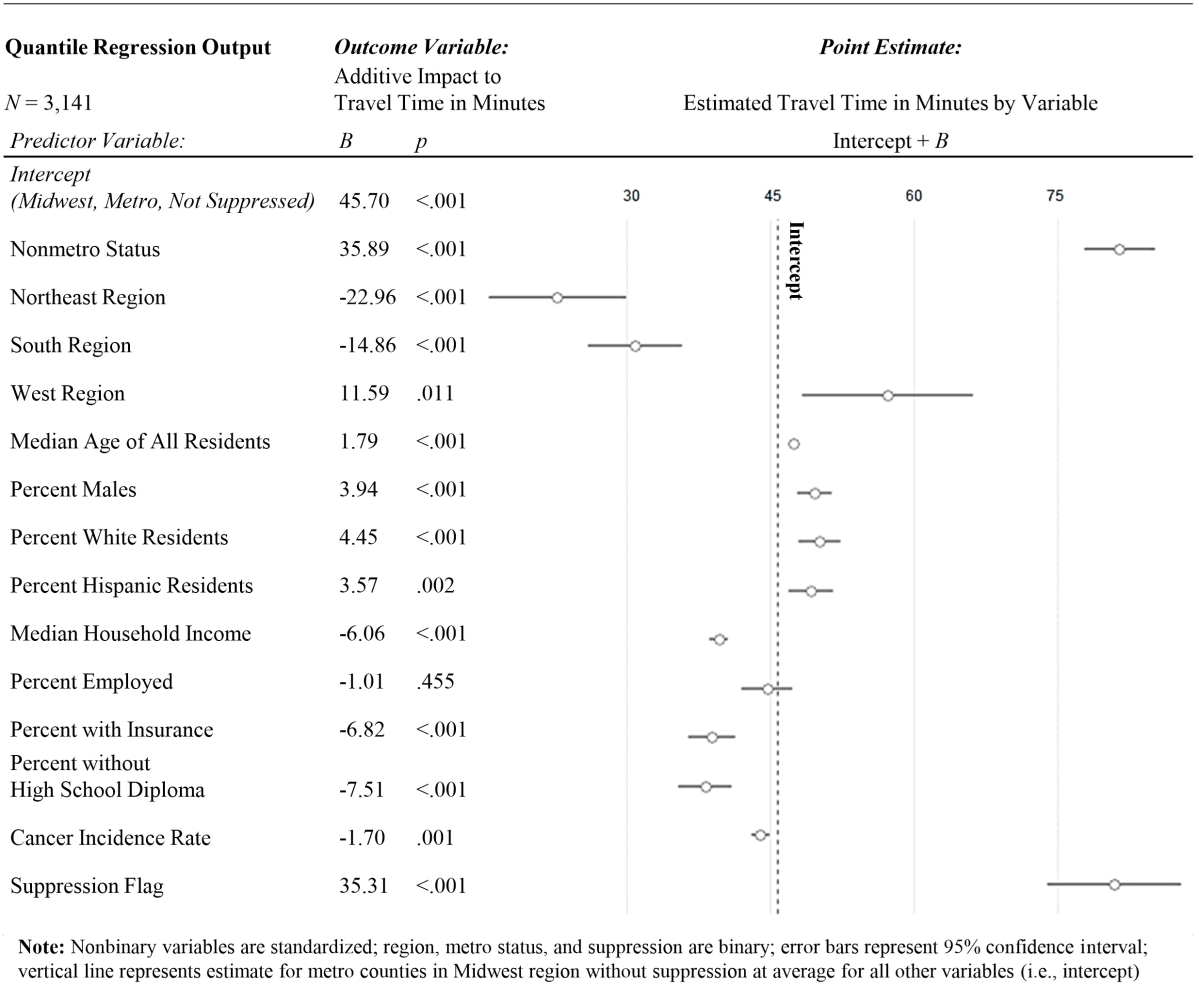
Quantile regression for factors associated with travel time to the nearest H&N specialist.

To determine the level of association between US region, metro vs non-metro status, social determinants of health, and travel time, a quantile regression model was evaluated (**Figure 4**). The alpha level used for significance was.05. In terms of geographic effects, Non-Metro status (*B* = 35.89, *p* <.001), Northeast region (*B* = -22.96, *p* <.001), South region (*B* = -14.86, *p* <.001), and West region (*B* = 11.59, *p* = .011) all differed statistically significantly from the reference group of Midwest Metro.

**Figure 5:**
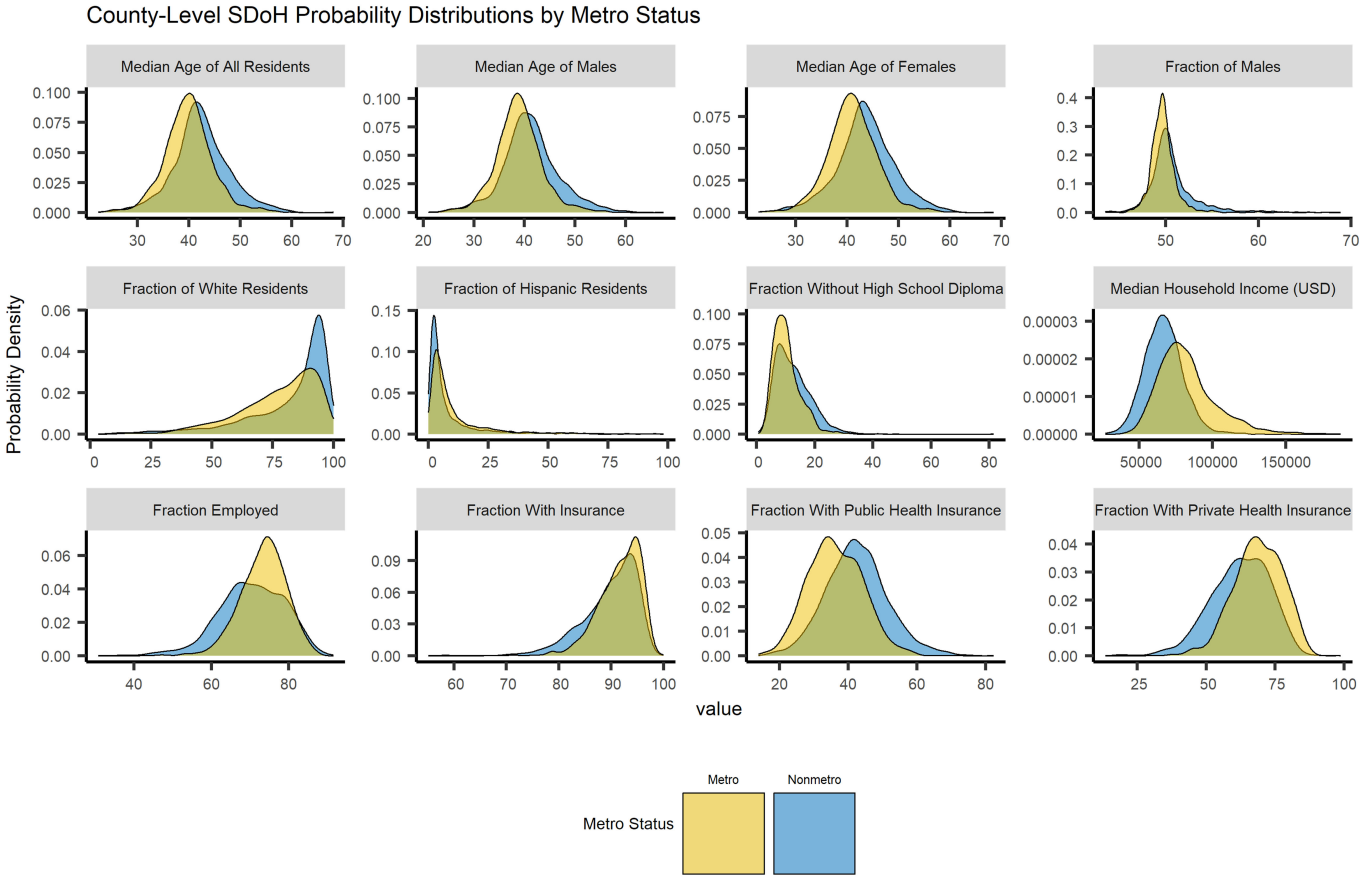
Social determinants of health by head and neck cancer specialist availability.

Among county-level demographic characteristics, age (*B* = 1.79, *p* <.001), percent male (*B* = 3.94, *p* <.001), percent white (*B* = 4.45, *p* <.001), and percent hispanic (*B* = 3.57, *p* = .002) all statistically significantly positively predicted travel time. Median household income (*B* = -6.06, *p* <.001), percent insured (*B* = -6.82, *p* <.001), percent without a high-school diploma (*B* = -7.51, *p* <.001), and cancer incidence (*B* = -1.7, *p* = .001) all statistically significantly negatively predicted travel time. Percent employed did not statistically significantly predict travel time. Counties with suppressed cancer incidence also differed statistically significantly positively from the comparison group (*B* = 35.31, *p* <.001). County-level demographics for counties with and without H&N specialists can be found in **Table 5**, and county-level SDOH probability density plots by provider coverage can be found in **Figure 5**.

**Table 5:** County demographics for counties with and without H&N specialists.

Characteristic (median by county)	Median for counties with H&N specialist (IQR^1^) *N = 277 counties*	Median for counties without H&N specialist (IQR) *N = 2,866 counties*	Wilcoxon Rank Sum Test with Bonferroni correction (*p*-value) *N = 12 tests*
Median age of all county residents in years	38.2 (4.4)	41.5 (5.9)	<.001
*Median age of male county residents*	37.1 (4.1)	40.3 (5.8)	<.001
*Median age of female county residents*	39.2 (4.8)	42.9 (6.5)	<.001
Fraction of county population that is male	0.492 (0.012)	0.500 (0.018)	<.001
Fraction of county population that is white	0.723 (0.222)	0.877 (0.193)	<.001
Fraction of county that is of Hispanic origin	0.097 (0.157)	0.044 (0.073)	<.001
Median household income in county in dollars	85,049 (25,109)	69,872 (18,928)	<.001
Fraction of people in county that are ages 16+ and employed	0.750 (0.479)	0.719 (0.107)	<.001
Fraction of people in county with any type of health insurance coverage	0.928 (0.056)	0.913 (0.064)	<.001
Fraction of people in county with public health insurance coverage	0.324 (0.10)	0.406 (0.118)	<.001
Fraction of people in county with private health insurance coverage	0.693 (0.115)	0.652 (0.144)	<.001
Fraction of people in county that are ages 25+ and without a high school degree	0.093 (0.046)	0.106 (0.074)	<.001

The predicted travel time for the Midwest Metro reference group was about 46 minutes, whereas travel times for non-metro regions were predicted to be over half an hour longer compared to metro regions (*B* = 35.89). Travel times in the Northeast and South regions were 22.96 minutes and 14.86 minutes shorter, respectively, compared to the Midwest. Older populations were statistically significantly further from care, with each year increase of median age for the county predicting 1.79 minutes of increased travel time. Counties with higher incomes and greater access to insurance were both found to be be statisticaly significantly closer to care. In terms of racial and ethnic demographics, counties with a higher percentage of males, white residents, hispanic residents and high school graduates were statistically significantly further from care, and counties with a higher cancer incidence rate tended to be closer to care.

### 3.3 Disparity index

**Figure 6:**
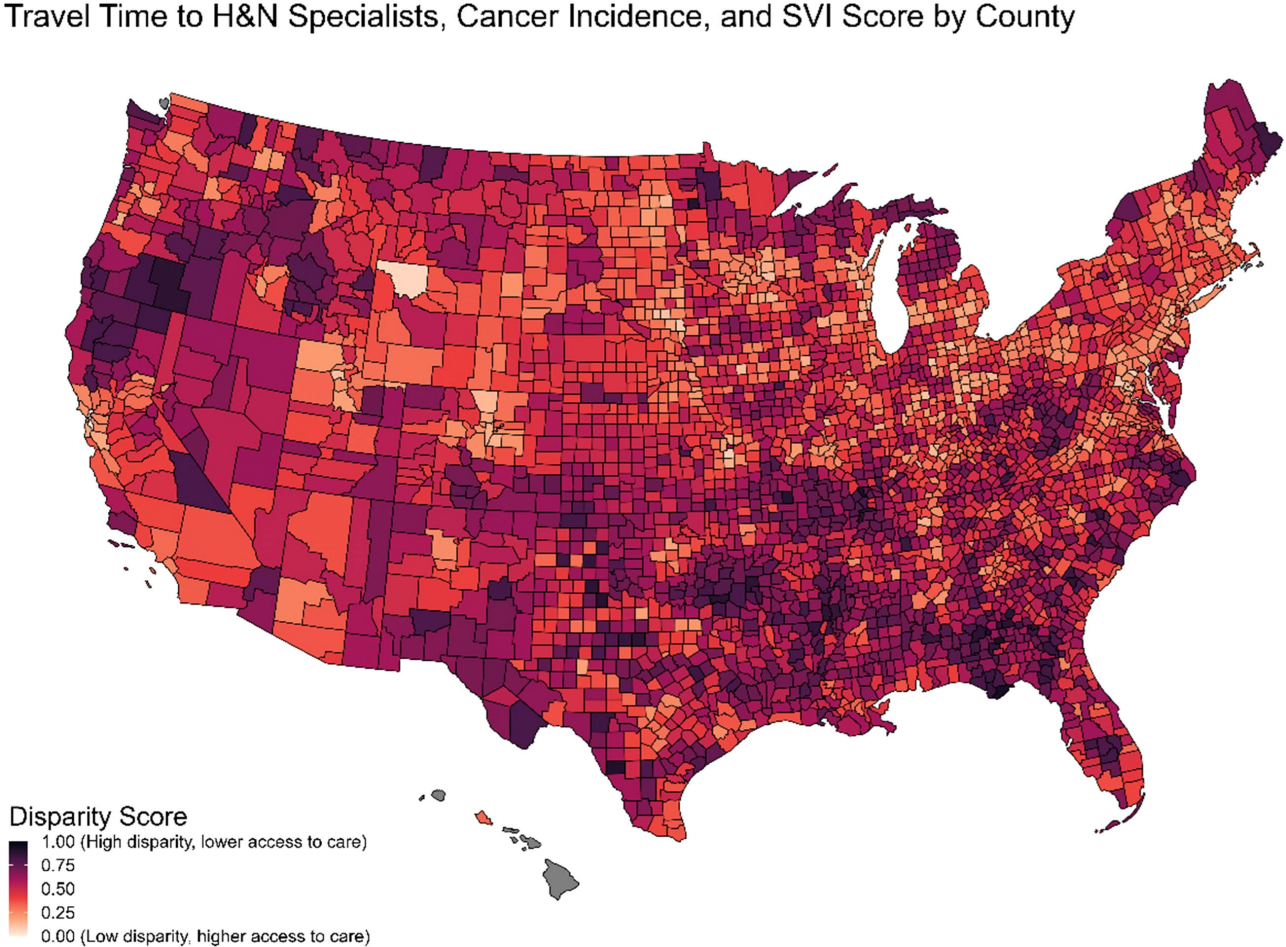
Disparity index from travel time, cancer incidence & social vulnerability index.

A disparity index (**Figure 6**) was computed using a linear combination of the county-level percentile rank of travel-time, cancer incidence, and social vulnerability (SVI). The calculated alpha-reliability for the index was 0.74.

**Table 4** displays the five highest and five lowest metro counties, and the five highest and five lowest nonmetro counties, by disparity index score. The states with the highest disparity index scores tend to be in the Southern region, whereas the states with the lowest disparity index scores tend to be in the Midwest region.

## 4 Discussion

Our study illustrates the differences in access to H&N specialist care throughout the US which, as predicted, is linked to socioeconomic status, racial and ethnic background, and healthcare infrastructure (20–24). Our research expands on prior reports by demonstrating the relative contributions of multiple county-level demographics and SDOH in access to specialized care, and by providing a tool in the form of the disparity index to highlight the areas of the US with the highest burden relative to their degree of access. Long travel times are known to impact the continuum of patient care, causing delayed diagnosis, reduced compliance, and limiting adherence to treatment, all of which are known to contribute to individual patient outcomes, reduced survival rates, and decreased quality of life (25, 29).

The analysis revealed significant regional disparities in healthcare access, with notably shorter travel time to H&N specialists in the Northeast compared to other regions. This discrepancy likely stems from higher urban density in the Northeast, leading to more concentrated healthcare facilities and more specialized cancer centers and academic hospitals, resulting in shorter travel distances for specialized care seekers. Infrastructure, such as transportation networks, may also contribute to this difference. Interventions to improve access in regions outside the Northeast could involve initiatives like mobile clinics and targeted outreach programs, as proposed by Moore et al. (27).

As expected, county-level analyses showed significant disparities between metro and non-metro areas in H&N specialist cancer care access, persisting after accounting for region and SDOH. Metro status consistently emerged as a prominent predictor of access disparity, explaining a substantial portion of outcome differences, aligning with existing literature (14, 25, 27).

Key SDOH indicators, such as family income, education, and transportation access, vary even within metro areas, underscoring the complexity of healthcare accessibility beyond urban-rural distinctions. These results show that median age and other county-level demographic characteristics, including the proportion of male, white, and Hispanic residents, all predicted longer travel times to the nearest H&N specialist, after controlling for metro status and geographic region. One potential explanation is that older populations may reside in areas with fewer specialized medical facilities, leading to increased travel times (32, 33). Similarly, counties with higher percentages of males and white residents might reflect demographic patterns associated with more rural or underserved areas, where specialist care is less accessible (14, 34). The higher travel times for counties with larger Hispanic populations could be linked to socio-economic disparities and potential geographic clustering in regions with limited healthcare infrastructure (24, 25, 34).

Results also showed that counties with higher household income, percent insured, and higher cancer incidence were associated with shorter travel times to the nearest head and neck cancer specialist. The positive impact of income and insurance coverage are intuitive as wealthier and insured populations are better equipped to access and afford specialist care and are therefore likely to have better healthcare infrastructure and more specialists, reducing the need for long travel (24, 33, 35, 36). Additionally, regions with higher cancer incidence rates may have more oncologists and specialized care facilities due to the increased demand for such services, thereby reducing travel distances for patients (37).

Counties with higher proportions of individuals lacking a high school diploma showed shorter travel times, aligning with existing research indicating that populations with no high school diploma often reside in urban areas with greater healthcare access, resulting in shorter travel times (32, 38). Potential additional explanations could also be that areas with lower educational attainment have invested in healthcare accessibility as a compensatory measure, there may be local community health initiatives aimed at improving access in these populations, or education might not uniformly influence healthcare accessibility across different regions, highlighting the need for further research to understand these complex dynamics (32, 37).

The Disparity Index, a novel metric, quantifies healthcare access gaps across US counties by considering geographical, socioeconomic, and epidemiological factors. These scores highlight areas where vulnerable populations face disproportionate challenges, which can potentially aid in decision-making about locations to establish additional H&N specialists fellowship programs. For example, a comparison between Alexander, Illinois and Scott, Illinois underscores significant differences in specialized care access despite similar geographic characteristics. The index identifies priority regions for policymakers and practitioners, guiding targeted resource allocation and advocating for interventions like mobile clinics. Strategic interventions, including incentivizing providers to serve underserved areas, can address high-disparity counties with limited specialist availability.

### 4.1 Limitations

The study's limitations, such as reliance on self-reported cancer specialization data and incomplete coverage including potentially outdated NPI records, suggest opportunities for future refinements, including supplementing sources with municipal or NGO data. Expanding into other cancer sites could improve estimation of specialist providers.

County-level data for SDOH has limitations; using more granular data like census tract or neighborhood level could provide better differentiation, especially in dense urban areas. Future investigations might benefit from exploring neighborhood-level data sourced from municipal databases, NGOs, or cancer registries to capture intricate urban variations accurately. Caution is warranted in assuming causation due to the observational nature of the analysis.

The Travel Time API could not provide estimated travel times greater than twelve hours, and so results for counties with an estimated travel time greater than twelve hours could not be included in the analysis. This limits the generalizability of the results, which may not be representative for counties above this threshold due to this technical limitation.

The unavailability of robust and geographically detailed treatment and mortality data is a limitation of this study. Future studies should incorporate geographically detailed cancer treatment and mortality data to enhance analysis. However, challenges such as variability in coroner and death certificate reporting practices may limit direct geographic comparisons of mortality data (39, 40).

### 4.2 Conclusions

The demonstrated disparities are known to contribute to health inequities (25–28), illustrating the pressing need for policy interventions to address health care access disparities, suggesting strategies such as incentivizing providers to work in underserved areas to promote equitable resource distribution. Advocacy groups could leverage these findings to promote policies, such as extending existing transportation programs or allocating resources. Clinicians and medical technology companies can utilize the disparity index to identify high-need areas, target outreach efforts, inform resource allocation decisions, improve patient education, and to design inclusive clinical studies, which may help mitigate the impact of the observed disparities (21, 27, 29).

The role of distance in accessing healthcare is critical, impacting patient decisions (25, 26). While this analysis assumed preference for the nearest care facility, future studies should explore distance's role in healthcare-seeking behaviors further and should also consider the role of telehealth in cancer care. Future studies should also explore disparities in care provided by generalists as opposed to specialists to understand its implications on healthcare access, as limited access to H&N specialists does not necessarily equate to limited access to cancer care overall.

In summary, this study highlights significant disparities in healthcare access, driven by geographical factors, metropolitan status, and social determinants of health. Urgent interventions are needed, urging policymakers to incentivize healthcare providers in underserved areas, clinicians to engage in patient education, and advocacy groups to raise awareness and advocate for equitable policies. Addressing these disparities will not only enhance access but also promote equitable healthcare provision for all individuals, irrespective of geographic or sociodemographic factors.

## Data Availability

Publicly available datasets were analyzed in this study. This data can be found here: ASCO (https://asco.org/), US News (https://health.usnews.com/doctors/), NPPES (https://download.cms.gov/nppes), SEER (https://seer.cancer.gov), USCS (https://www.cdc.gov/cancer/uscs), the US Census (https://www.census.gov/data.html), and the CDC (https://www.atsdr.cdc.gov/placeandhealth/svi/). Additional cancer incidence epidemiology data that were unavailable in SEER and USCS were obtained directly from state cancer registries in Nevada, Minnesota, Virginia, and Kansas.
